# Cross-cultural differences in somatic awareness and interoceptive accuracy: a review of the literature and directions for future research

**DOI:** 10.3389/fpsyg.2014.01379

**Published:** 2014-12-03

**Authors:** Christine Ma-Kellams

**Affiliations:** Department of Psychology, University of La VerneLa Verne, CA, USA

**Keywords:** culture, somatic awareness, interoception, somatization, meditation

## Abstract

This review examines cross-cultural differences in interoception and the role of culturally bound epistemologies, historical traditions, and contemplative practices to assess four aspects of culture and interoception: (1) the extent to which members from Western and non-Western cultural groups exhibit differential levels of interoceptive accuracy and somatic awareness; (2) the mechanistic origins that can explain these cultural differences, (3) culturally bound behavioral practices that have been empirically shown to affect interoception, and (4) consequences for culturally bound psychopathologies. The following outlines the scope of the scientific review. Part 1 reviews studies on cultural variation in spontaneous somatic word use, linguistic expressions, traditional medical practices, and empirical laboratory studies to assess the evidence for cultural differences in somatic processes. Integration of these findings suggests a startling paradox: on the one hand, non-Western cultures consistently exhibit heightened somatic focus and awareness across a variety of contexts; on the other hand, non-Western cultures also exhibit less interoceptive accuracy in laboratory studies. Part 2 discusses the various mechanistic explanations that have been proposed to explain these cultural differences in somatic awareness and interoceptive accuracy, focusing on cultural schemas and epistemologies. Part 3 addresses the behavioral and contemplative practices that have been proposed as possible “interventions,” or methods of cultivating bodily awareness and perceptual accuracy. Finally, Part 4 reviews the consequences of interoception for psychopathology, including somatization, body dysmorphia, eating disorders, and anxiety disorders.

How does culture shape interoception? A growing body of psychological research has compellingly demonstrated that humans are a uniquely cultural species insofar as the extent to which we are able to learn from our social groups and the extent to which such learning can radically change a broad spectrum of our thoughts, feelings, and behaviors (Tomasello, 1999; Richerson and Boyd, 2005; Heine and Norenzayan, 2006). As a result of these advances, many of the most foundational psychological processes previously assumed to be universal have been shown to be profoundly culturally bound (for review, see Heine and Norenzayan, 2006).

Although this notion that culture shapes our most basic features of our psyche has been applied to a host of processes, there has been few attempts to systematically understand how cultural forces shape awareness of the body. Thus, the purpose of this review is to elucidate the role of culturally bound epistemologies, historical traditions, and contemplative practices in shaping somatic awareness and interoceptive accuracy. Given the multitude of ways that a process as complex as interoception and culture can be studied, the approach here will be interdisciplinary—bringing together evidence from anthropology, clinical science, and psychology.

Specifically, the review will focus on four key features of culture and interoception. First, I will review both empirical and ethnographic evidence that examines the extent to which members from Western and non-Western cultural groups exhibit differential levels of somatic awareness and interoceptive accuracy (Part 1). From there, I will discuss the proposed mechanistic origins that can explain these cultural differences (Part 2). I will then review culturally bound behavioral practices that have been empirically shown to affect interception (Part 3), and end with a discussion for how culturally bound interoception many have consequences for culturally bound psychopathologies (Part 4).

## PART 1: CROSS-CULTURAL DIFFERENCES IN SOMATIC AWARENESS AND INTEROCEPTIVE ACCURACY

As with virtually all scientific inquiry, the first step to understanding a complex phenomena is to predict and discover interesting features of the process at hand (see Cronbach, 1986; Rozin, 2001, for review). In the context of cultural processes and interoception, this first stage involves identifying key cultural differences in the way individuals perceive and understand their own bodies. To what extent do people from different cultural backgrounds vary in their somatic awareness and interoceptive accuracy?

The question of how cultures differ in somatic awareness and interoceptive accuracy is a challenge because the two process, albeit related, may not align insofar as whether or not cultural differences in the one also entails (the same) cultural differences in the other. Somatic or interoceptive awareness centers on the extent to which individuals find bodily cues salient, whereas interoceptive accuracy centers on individuals’ abilities to accurately infer the cause and magnitude of their bodily changes. While interoceptive awareness is typically operationalized as the frequency of reporting bodily sensation and beliefs about the importance of such bodily states, interoceptive accuracy is typically operationalized as the degree of precision in reporting actual bodily states (e.g., heart rate). This distinction is also reflected on the neural level, as somatic awareness and interoceptive accuracy rely on different brain mechanisms (Critchley et al., 2004). Somatic awareness stands as a firmly top–down process that is driven by attention, beliefs and expectations (Rimé et al., 1990; Philippot and Rimé, 1997). In contrast, interoceptive accuracy depends on both bottom–up processes (e.g., the detection of bodily cues and the presence of physiological features that facilitate such detection, such as fitness level—Jones et al., 1987), as well as top–down ones (e.g., attention, cultural schemas—Pennebaker and Hoover, 1984; Van den Bergh et al., 1998; Wiens, 2005). Thus, the former does not serve as simply a proxy for the latter. Indeed, outside the cultural literature, a number of studies have found that awareness and accuracy can be dissociated (Pennebaker and Hoover, 1984; Gardner et al., 1990; Pennebaker, 1995; Critchley et al., 2004), or even inversely related (Fairclough and Goodwin, 2007). Given their qualitative differences, evidence for cultural differences in each process will be reviewed separately, and their implications will subsequently be discussed.

These challenges in disentangling somatic awareness from interoceptive accuracy are further complicated by the fact that somatic awareness is, in itself, a multi-faceted construct that have been construed in different ways across different disciplines (for review, see Mehling et al., 2012). On the one hand, somatic awareness can be defined by the outcome achieved by focusing direct attention on in-the-moment bodily changes and affective responses; training aimed at increasing this type of awareness (e.g., concrete somatic monitoring; sensory discrimination) suggests that it can be adaptive (Flor et al., 2001; Watkins and Moulds, 2005). In contrast, ruminating on the body for the purpose of vigilance appears to be a less adaptive form of somatic awareness (Cioffi, 1991; Cioffi and Holloway, 1993; Watkins and Moulds, 2005). Additionally, a further distinction can be made between proprioceptive versus interoceptive awareness. While the former centers on perception of muscles, joints, movements, posture, and balance (Laskowski, 2000), the later centers on perception of internal bodily sensation—including, but not limited to: heart rate, breathing, and hunger (Vaitl, 1996; Cameron, 2001; Craig, 2002; Barrett et al., 2004).

Given this multi-dimensional nature of somatic awareness, it is difficult to draw conclusive inferences about the nature of cross-cultural variation in how people perceive bodily states. Nevertheless, convergent evidence from both empirical studies and ethnographic work is suggestive that members of a number of non-Western cultures may exhibit higher levels of somatic awareness than members of Western cultures. In their seminal work on culture, emotion, and language, Tsai et al. (2004) found that Chinese–Americans respondents consistently used more somatic words than European–Americans when discussing a variety of events, including their relationships, early childhood experiences, and conversations with their romantic partners; furthermore, even among Chinese–Americans, those who were less acculturated to North-American culture exhibited this greater reliance on somatic words relative to those who were more acculturated. This finding is consistent with a broad body of work that has suggested that Chinese culture perceives bodily and psychological states to be closely intertwined (Kleinman, 1986; Ots, 1990). Likewise, close examination of the findings on culture and the self from Kanagawa et al. (2001) study on spontaneous self-description among college students highlights the disproportionately higher use of somatic descriptors among Japanese compared to North Americans. Taken together, these findings suggest that East-Asians appear to demonstrate a greater emphasis on their bodily states when describing themselves and their emotional experiences.

Additional evidence from linguistics suggests that this greater emphasis on somatic cues in everyday life among East-Asians may be traced back to historic traditions steeped within Asian language and medicine. In the Chinese language, for example, many idiomatic emotional expressions use the body parts (specifically, visceral organs) as metaphors: xuan-xin diao dan (to have one’s heart in one’s mouth), ti xin diao dan (lift the heart, hang the gallbladder), dan-zhan xin jib (gall trembling, heart startled), dan-po xin jin (gall breaking, heart startled), jing-xin diao-dan (shock the heart, drop the gallbladder), xin-dan ju lie (heart and gallbladder, both split), all refer to states of fear, where the heart and the gallbladder are described as lifted, hanging in the air, jolted, broken, dropped, or torn. We find the same pattern of somatic focus in Chinese holistic medicine, which model the body as a powerful psychological force. According to traditional holistic models of the human body, decision-making and thought reside primarily not in the mind, but in the liver, the site of contemplation; the gallbladder, the place where judgments are made; and the heart, the executive center (Ye, 2002).

Similar cultural patterns also emerge in other East-Asian cultures. In Japanese, anger starts off by being contained the belly (hara), then progresses to the chest (mune), and finally reaches the head (atama; Matsuki, 1995). Given that cultural ideas and language have long been argued to be inseparable—after all, language stands as the primary mechanism for transmitting cultural ideas (Wierzbicka, 1993, 1995; Tomasello, 1999; Slobin, 2003)—these provide convergent evidence of cross-cultural variation in the degree to which individuals emphasize somatic experiences.

East-Asians cultures are not the only non-Western cultural groups that exhibit this pattern. A growing number of recent studies has found that many cultural groups, including those from Papua New Guinea (Lindström, 2002), aboriginal Australia (Turpin, 2002), and West Africa (Geurts, 2003) display a similar emphasis on the body. In the West African case, anthropologists have observed that cultural terms exist that refer exclusively to bodily sensations for which there is no English translation (e.g., “seselelame,” which can be roughly translated as “feel-feel-inside-the-body”—Geurts, 2003). Likewise, many West-African languages use the body as a basis for their emotional terms (Ameka, 2001; Dzokoto and Okazaki, 2006), much like the way they are used in Chinese and Japanese. Likewise, African traditional medicine also focuses on harmony and holism, treating the body and mind as fundamentally integrated rather than separate (Mbiti, 1970). In line with these cultural practices, it is not surprising that recent research by Dzokoto (2010) and Chentsova-Dutton and Dzokoto (2014) found that West-Africans also self-reported greater sensitivity to bodily changes relative to European–Americans.

Taken together, these findings offer suggestive—but not definitive—evidence for cultural variation in somatic awareness. At best, the majority of the aforementioned work has yielded indirect evidence for a greater cultural emphasis on bodily parts and processes among members of non-Western cultural groups—namely, East-Asians and West-Africans. Given the indirect nature of this evidence, it remains unclear the precise nature and form of these cross-cultural differences, and whether they extend to proprioception or interoception, mindful awareness of bodily states or vigilant monitoring of bodily cues.

These limitations notwithstanding, to what extent does this potential cultural difference in somatic awareness translate into interoceptive accuracy? As a construct, interoceptive accuracy stands as a crucial feature of numerous emotion theories (e.g., James, 1884; Schachter and Singer, 1962; Damasio, 1994) with a wide range of consequences for a variety of psychological processes, including self-regulation (e.g., Barrett et al., 2004), decision-making (e.g., Werner et al., 2009) and attention (e.g., Matthias et al., 2009). Although the majority of studies to date on interoceptive accuracy have focused on individuals’ abilities to accurately detect cardiac signals given its non-invasive nature, more recent findings suggest that cardiac accuracy predicts other forms of accuracy—namely, sensitivity for gastric functions, and thus may reliably stand as a more general indicator of interoceptive accuracy (Herbert et al., 2012b). Furthermore, a number of individual differences, situational forces and practices can influence interoceptive accuracy, including: food deprivation (e.g., Herbert et al., 2011), gender (e.g., Koch and Pollatos, 2014a); obesity (e.g., Herbert and Pollatos, 2014), and disordered eating (e.g., Koch and Pollatos, 2014b).

A striking paradox emerges in the literature on culture, somatic awareness, and interoceptive accuracy. Until now, few studies have dealt specifically with the relationship between culture and internal bodily states. The bulk of the existing literature has focused exclusively on actual (rather than perceived) bodily changes and has found few, if any, cultural differences (Tsai et al., 2002; Soto et al., 2005). Thus, it appears that there is little evidence for consistent cross-cultural variation in actual bodily events.

Nevertheless, recent work by Ma-Kellams et al. (2012) found that when it comes to perceiving bodily changes, East-Asians are less accurate than European–Americans. Across four studies, East-Asians consistently demonstrated less interoceptive accuracy: they were more likely to misattribute the cause of their bodily changes and displayed greater discrepancies between their perceived and actual bodily states (in this case, heart rate).

Chentsova-Dutton and Dzokoto (2014) found a similar pattern of results with West-Africans. Despite the fact that West-Africans reported higher levels of interoceptive awareness, they nevertheless displayed less interoceptive accuracy—as in the previous case, they were less able to accurately report their heart rate relative to European–Americans. Taken together, these two studies offer limited and tentative evidence for an interesting paradox: both East-Asians and West-Africans are simultaneously more aware of their own bodies (“aware” insofar as they find bodily features more salient in everyday life and report higher levels of somatic sensitivity), and yet they display a relative inability to accurately infer bodily changes. How can this be?

## PART 2: EXPLAINING CROSS-CULTURAL VARIATION IN SOMATIC AWARENESS AND INTEROCEPTIVE ACCURACY

Part 1 of this review, in identifying the key differences in somatic focus and interoceptive accuracy between Western and non-Western cultural groups, highlighted an important puzzle: members of East-Asian and West-African cultures appear to be both more somatically focused and more inaccurate in their somatic inferences. This raises the deeper issue of how the previously observed cultural differences in interoception can by explained. In the following sections, I articulate the divergent proposed explanations for these cultural differences in somatic focus and interoceptive accuracy.

Given that culture, at its core, consists of collectively shared meaning systems that involve beliefs, values, language, and rituals that serve to both produce behavior in culturally consistent way and reinforce such behavior (Kroeber and Kluckhohn, 1952), it is not surprisingly that the long-standing explanation put forth for why cultures vary in the degree of somatic focus is differences in these culturally specific meaning systems. However, there lacks a consensus as to which feature of culture is the critical one that can explain differences in bodily focus. Some scholars argue that differences in cultural conceptualizations of the body and how it relates to other key features of the self is the primary process at hand; namely, Eastern cultural models of the self and emotion portray the body as fundamentally entwined with the body (Kleinman, 1986; Ots, 1990). Other scholars, in contrast, contend that differences in language is the critical driving force, and that for members of a Chinese-speaking culture, for example, somatic and emotion words are less differentiated (i.e., bodily words are oftentimes embedded in other words—Tung, 1994).

Work by Tsai et al. (2004) addresses this question of mechanism by dissociating cultural conceptions from language. In comparing European–Americans, less-acculturated Chinese–Americans, and more-acculturated Chinese–Americans speaking the same language (i.e., English), they were able to directly contrast the effects of cultural beliefs while holding language constant. If differences in somatic focus between members of Eastern and Western cultures were the product of language differences, then their experimental design would have yielded no differences between the three cultural groups given that all spoke the same (English) language. If somatic focus differences were the product of culturally specific conceptions about the psychological meaning afforded to bodily processes, then differences between the three groups would appear, despite the use of shared language. Their findings provide support for the latter model, as less-acculturated Chinese–Americans displayed more somatic word use relative to more acculturated Chinese–Americans and European–Americans.

Similar arguments have been made to explain West-Africans’ greater somatic awareness. Chentsova-Dutton and Dzokoto (2014) argued that top–down factors like cultural schemata surrounding the role of the body, their accompanying culturally specific terms (e.g., “seselelame”), and their use of emotional expressions that integrate body parts (e.g., fear as “heart-fly”) can explain West-African’s self-reported sensitivity to bodily cues.

In contrast to this focus on cultural conceptions of the body used to explain cross-cultural variation in somatic focus, a different set of mechanisms have been used to explain differences in interoceptive accuracy. Given that cultural differences in these two processes diverge (i.e., with non-Westerners being more likely to display the former but less likely to display the latter), it is not surprising that attempts to explain East-Asians and West-Africans’ relative inaccuracy in interoceptive perception have focused not on cultural models of the body, but a different set of processes that are more cognitive in nature.

Attempts to explain cultural variation in interoceptive accuracy between East-Asians and European–Americans have focused on analyses of East–West epistemologies, and have suggested that despite the heightened focus on bodily processes, cultural differences in cognitive styles may nevertheless render those from Eastern cultures less able to accurately attend to internal cues. Specifically, Ma-Kellams et al. (2012) proposed that Asians and European–Americans differ in interoceptive abilities due to differences in context dependency. Past research on culture and cognition has consistently demonstrated that Easterners attend more to contextual cues when evaluating both the self (e.g., Kanagawa et al., 2001) and others and external events (e.g., Morris and Peng, 1994). Furthermore, such an attentional difference appears to be more than a matter of voluntary control: even when asked to ignore contextual cues, Asians exhibit greater difficulty (compared with European–Americans) on such tasks (Ji et al., 2000; Masuda and Nisbett, 2001; Kitayama and Ishii, 2002; Ishii et al., 2003; Kitayama et al., 2003). This focus on contextual cues should render Asians less attentive to their internal states (relative to the external cues stemming from the external world) because if the individual self is not the central object of focus or primary unit of analysis compared to the surrounding context, then bodily changes should be relatively less attended to. In other words, Asians tend to disproportionately focus on external contextual entities outside of themselves—both in terms of other individuals (as part of the interdependent self—Markus and Kitayama, 1991) as well as other factors in their environment (e.g., field-dependence—Ji et al., 2000)—and yet accurate interoception requires one to ignore such external factors in order to focus on one’s internal state. Thus, the argument is that contextual dependency explains East Asians’ lower interoceptive accuracy. Consistent with this argument, Ma-Kellams et al. (2012) found that individual differences in the ability to ignore contextual cues mediated performance differences between Easterners and Westerners on an interoceptive task—in this case, heartbeat detection.

In the West-African cultural context, Chentsova-Dutton and Dzokoto (2014) proposed that the higher levels of somatic awareness reported by members of this cultural group may be the precise mechanisms that hinders their interoceptive accuracy. That is, West-African culture holds a particular schema that links fear with a racing heart. Although this schema may accurately portray the link between emotion and physiological change in general, it may not serve to accurately describe online (i.e., in-the-moment) bodily changes. Thus, in the context of their study, in which they had West-Africans estimate their own heart rates while watching a fear-inducing film, it might have been possible that the saliency of the emotional content led these participants to expect increases in heart rate (in line with the schema) but ignore the actual, more subtle cues from their body. Thus, another possible explanation for cultural differences in interoceptive accuracy is that cultures that exhibit high levels of somatic focus may ironically be worse at detecting actual somatic change because highly salient somatic schema are chronically accessible and renders individuals less likely to attend to actual somatic cues.

## PART 3: CULTURAL PRACTICES AND INTEROCEPTION

Culture is not known of its timeless, unchanging nature; if anything, culture changes as social life changes, and a fundamental component of culture is the practices, rituals, and traditions individuals of any given cultural group rely on that promotes culturally consistent ways of thinking, feeling, and behaving. Thus, this section focuses on the behavioral and contemplative practices from different cultural origins that have been proposed as possible “interventions,” or methods of cultivating bodily awareness and perceptual accuracy.

Meditation, mindfulness, and yoga are ancient Eastern practices, but in recent years they have received increasing empirical attention in the context of psychological well-being and health. At the core, yoga and its related practices takes a holistic approach to the mind and body, assuming that exercises with a mental focus will have bodily effects, and bodily exercises will have mental effects. Together, they share a variety of common features, including prolonged physical stillness and/or some kind of mental control characterized by stability and focus, a perceptual style in which there is little active effort to interpret sensory information. From a scientific standpoint, they have conceptualized these practices as a complex set of emotional and attentional training regimens (Lutz et al., 2007). Although these techniques are traditionally used in spiritual contexts, there is a growing trend in using them as a form of alternative therapy (Astin et al., 2003; Barnes et al., 2004; Arias et al., 2006).

Meditation, yoga, and mindfulness techniques typically incorporate somatic awareness and use the body as an object of focus (Kabat-Zinn, 1990; Selby, 1992; Kornfield, 1996; Nairn, 2000). It is important to note that in this context, somatic awareness is defined primarily by adaptively focusing direct attention on in-the-moment bodily changes (Flor et al., 2001; Watkins and Moulds, 2005) rather than engaging in emotionally driven vigilance (Cioffi, 1991; Cioffi and Holloway, 1993; Watkins and Moulds, 2005).

Beyond focusing on the body, bodily sensations are also modulated through breathing and posture (Bhajan and Khalsa, 2000; Arambula et al., 2001; Peng et al., 2004). Theorists have posited that the benefits observed from practicing any or all of these actions are derived from these common elements (for review, see Watts, 2000), and followers of these practices attest that engaging in this kind of intentional attunement leads to improved awareness of a variety of internal states, including bodily awareness (Kabat-Zinn, 1990; Kornfield, 1996; Nairn, 2000).

Khalsa et al. (2008) empirically tested this assertion by examining interoceptive accuracy among two groups of experienced meditators, Tibetan Buddhists and Kundalini monks, and comparing them to a group of non-meditators. The authors assessed both self-perceived interoceptive awareness (i.e., participants’ reports of their interoceptive performance) and actual interoceptive accuracy (i.e., on a heartbeat detection task). Contrary to prediction, experienced meditators displayed comparable levels of accuracy relative to non-meditators, but self-reported higher levels of interoceptive awareness. Similar findings have also been reported by Nielsen and Kaszniak (2006), albeit with a smaller sample size and no control group. Taken together, these findings suggest that contrary to lay assumptions, chronic engagement in meditative practices only appears to heighten somatic awareness but does not appear to improve actual interoceptive accuracy.

Despite the lack of evidence for a direct link between meditative practices and interoceptive accuracy, these practices are not without other physiological and psychological benefits. Empirical studies comparing yoga with relaxation exercises, for example, have found that both practices yielded similar physical and psychological benefits (e.g., decreases in heart rate and blood pressure, increases in self-esteem—Jaggi, 1979; Cusumano and Robinson, 1992). Other studies have found additional physiological benefits of yoga, including asthma and hyperventilation (Chandra, 1994) and hypertension (Steptoe, 1981). Similar arguments have been made about mindfulness techniques, which have been shown to prevent depressive rumination (e.g., Teasdale et al., 1995). Recently, Shannahoff-Khalsa proposed that combining meditation with breathing and somatic exercises based on the Hindu Tantric practice of Kundalini Yoga can be used as an intervention for a wide range of psychiatric disorders. Thus, a review of the empirical studies on meditation, mindfulness, and yoga reveals mixed evidence for the effects of such contemplative practices on bodily awareness and interoceptive accuracy—these meditative practices appear to facilitate the former, but not the latter; these limitations notwithstanding, they may offer physiological and psychological benefits in other contexts apart from interoception.

## PART 4: CULTURE, INTEROCEPTION, AND PSYCHOPATHOLOGY

A substantive body of work demonstrates that members of different cultural backgrounds exhibit differential levels of somatic symptoms in response to both physical and mental illness. In the context of physical illness, African–Americans are more likely to report bodily symptoms after exercise, surgery, and in response to a variety of diseases (Faucett et al., 1994; Sheffield et al., 1999; Edwards et al., 2001). In the context of psychological distress, members of non-Western cultures are more likely to report bodily symptoms rather than purely affective ones; this pattern has been demonstrated with African (Chowdhury, 1996; Dzokoto and Adams, 2005); African–American (Friedman and Paradis, 2002); Cambodian (Hinton et al., 2006, 2007); and Chinese samples (Park and Hinton, 2002; Ryder et al., 2008; Ryder and Chentsova-Dutton, 2012).

The heightened rates of somatization of psychological distress in Asian culture is perhaps the most widely studied example of cultural variation in the cultural psychopathology literature (see Ryder et al., 2002). Compared to individuals of European descent, those from Asian countries are allegedly more likely to manifest bodily symptoms when experiencing psychological distress. In one of the initial studies on culture and epidemiology, Kleinman and Good (1985) found that depression was rarely reported in Chinese cultures, but neurasthenia—a similar illness characterized by somatic symptoms—was much more prevalent; he subsequently concluded the neurasthenia emerged as a culturally specific manifestation of depression. Subsequent studies have similarly found a greater tendency among Chinese to report somatic symptoms (Tsoi, 1985; Chan, 1990; Simon et al., 1999; Yen et al., 2000; Parker et al., 2001). In the attempt to explain these cultural differences, numerous theoretical arguments have been put forth, including linguistic features of the Chinese language (e.g., Leff, 1981); stigma associated with psychiatric conditions (e.g., Goffman, 1963), and differences in emotional expression norms (Sayar et al., 2003).

More recent research has challenged both theoretically and empirically. Theoretically speaking, Cheung (1995) posited that most of the explanations put forth for cultural variation in somatization were formed on a *post hoc* basis rather than built as part of the study designs. Empirically, Ryder et al. (2008) contended that many of the existing studies that have found cultural differences in somatic symptom reporting lacked a Western comparison sample and thus could not rule out the alternative explanation that somatic symptoms is a general feature of depression; furthermore, most studies relied on a single assessment mode, thus leaving the influence of modality on the finding an open question. Thus, in their study, Ryder et al. (2008) used three different assessment modalities (self-report, clinical interview, questionnaire) to assess symptom presentation among Chinese and European–Canadians; they found that although Chinese patients reported more somatic symptoms than European–Canadians, European–Canadians reported more psychological symptoms, and the latter effects was larger and more consistent than the former. The authors concluded that in the context of depression, cultural differences may center more on Western “psychologization” rather than Eastern somatization.

Zaroff et al. (2012) also challenged the long-standing view of somatization as a culture-specific pathology in their review of the literature, which found that rates of somatic symptom reporting are comparable across cultures when ascertainment methods are controlled for. They suggest that cultural variation in stigma associated with psychological illness and service provision, along with cultural socialization patterns, can explain what appears to be cultural differences in symptom reporting; however, when assessment techniques include direct questioning of mood and consider response patterns on self-report, much of the aforementioned cultural differences are attenuated.

Beyond depression, limited work has examined cultural differences in somatization with other psychopathologies. For example, Viernes et al. (2007) examined the link between somatization of distress, food restriction and fat phobia among Filipino and Western teenagers. They found that Western teenagers displayed more food restriction practices and fat phobia, and lower levels of somatization of distress. Fear of fatness was correlated with somatization, and the authors raised the question of whether fat phobia and somatization stand as culturally specific forms of expressing psychological distress (see also Helman, 1990). Nevertheless, it remained unclear based on their data whether such a link was causal or epiphenomenal. However, given that past studies have reliably shown interoceptive accuracy to relate to food deprivation (Herbert et al., 2011, 2012a), obesity (Herbert and Pollatos, 2014) and disordered eating (Koch and Pollatos, 2014b), it is likely that cultural differences in accuracy may also lead to related differences in food and eating disorders.

Likewise, somatization has been linked to other disorders in cultural contexts, including a variety of anxiety-based disorders (e.g., ataque de nervios, or “attack of nerves,” an unexplained distress syndrome found in parts of Latin-America—Lopez et al., 2011; Dhat syndrome, a culturally bound preoccupation with perceived semen loss found in India—Ranjith and Mohan, 2006; “Ode Ori,” a disorder characterized by a perceived crawling sensation in the body found in Nigeria—Makanjuola, 1987). Beyond these culture-specific disorders, somatic symptoms has also been linked to more generalized reports of anxiety and depression (e.g., in Pakistan—Minhas and Nizami, 2006; Israel—Al-Krenawi and Graham, 2004; Egypt— Abdel-Khalek and Lester, 2009; Mexico—Varela et al., 2004), as well as specific reports of post-traumatic stress and panic disorder (e.g., in Cambodia—Boehnlein, 2001). Though suggestive, the limited number of studies done on the role of culture and somatization in these disorders make it difficult to draw firm conclusions about how and why somatic symptoms feature so prominently in a variety of both generalized and culture-specific anxiety-based and depression-based disorders. Nevertheless, the prevalence of somatization in non-Western cultures is largely consistent with the finding that many non-Western cultural group members exhibit lower levels of interoceptive accuracy, given that somatization may reflect an inability to accurately perceive one’s bodily states. Indeed, a growing body of research supports this notion that somatization is a reflection of poor interoceptive abilities (Gardner et al., 1990; Bogaerts et al., 2008).

## CONCLUSION

In summary, a review of the existing literature suggests that cross-cultural differences in interoception can be summarized as follows: (1) members of non-Western cultures tend to exhibit higher levels of somatic awareness but lower levels of interoceptive accuracy; (2) variation in cultural conceptualizations and epistemic traditions can, in part, explain these differences, (3) cultural practices related to meditation, yoga, and mindfulness, in line with the aforementioned evidence, appear to facilitate bodily awareness but fail to improve actual accuracy, and (4) the heightened somatic awareness among non-Western cultures is linked to a greater emphasis on somatic symptoms in a wide array of psychopathologies—most notably, depression and anxiety.

These findings notwithstanding, there remains several areas that warrant further research in the context of culture and interoception. First and most broadly, the overwhelming majority of research in culture and interoception in particular and cultural psychology in general has been focused on the East–West comparison. Although this reliance on comparing members of East–Asian and European–American cultures has been fruitful and telling, there remains a substantive gap in our knowledge about how Western models of embodiment and related psychological processes emerge—or fail to emerge—in other cultural contexts. A small but growing body of work has begun to tackle this question in West Africa and Latin-America, but there remains much to be explored in other cultural contexts.

A related need is greater precision in defining the nature of observed cross-cultural differences. Few studies, if any, have attempted to take a multidimensional view of somatic awareness when assessing for cross-cultural differences. However, given recent developments in our understanding of the complex nature of body awareness (Mehling et al., 2012), future research can more systematically use multidimensional measures of somatic awareness (e.g., the MAIA—Mehling et al., 2012) in cross-cultural contexts. Similarly, more work is needed to assess the robustness of cross-cultural differences in interoceptive accuracy given the relative paucity of empirical studies to date that have investigated this construct.

Second, little is known about the role of somatic awareness and interoceptive accuracy in cross-cultural, psychopathological contexts apart from those relating to depression and/or anxiety. An example of one pathology that warrants further research is the role of culture and interoception in eating disorders. Although there is some initial evidence that interoception is a key process in anorexia nervosa (e.g., Arnold, 2012) and rates of anorexia nervosa varies across cultures (e.g., see Simpson, 2002, for review), few studies, if any, have examined whether interoceptive differences can explain cultural variation in this disorder. The case of anorexia nervosa serves as just one example of a psychopathology that may be moderated by culture and interoception, but for which there is limited research.

Third, more research is needed to elucidate how and when culture-specific practices—including, but not limited to mindfulness, meditation, and yoga—can assist in or hinder interoceptive accuracy. Despite the fact that existing empirical studies have failed to find a reliable relationship between experienced meditators and interoceptive accuracy, various alternative explanations remain. Subsequent studies can aim to manipulate the practice of meditation rather than rely on a self-selected sample of existing meditators to more directly assess the causal link between meditation and interoceptive accuracy. Furthermore, additional studies can assess interoceptive abilities in a wider array of forms (e.g., in modalities other than heartbeat detection) and bodily states (e.g., in states other than resting state). Doing so can help further elucidate the precise mechanisms and boundary conditions underlying the relationship between cultural practices and interoception.

## Conflict of Interest Statement

The author declares that the research was conducted in the absence of any commercial or financial relationships that could be construed as a potential conflict of interest.
