# Addendum: Cross-cultural differences in somatic awareness and interoceptive accuracy: a review of the literature and directions for future research

**DOI:** 10.3389/fpsyg.2016.01240

**Published:** 2016-08-17

**Authors:** Christine Ma-Kellams

**Affiliations:** Department of Psychology, University of La VerneLa Verne, CA, USA

**Keywords:** culture, somatic awareness, interoception, somatization, meditation

Reason for addendum:

In the original version of this paper, parts of the text inadvertently contained notes and overlooked important contributions from the literature, resulting in statements that were improperly worded or insufficiently cited. Below are the corrections, with apologies to the authors.

In the original text in Part 1: Cross-Cultural Differences describing the differences between somatic or interoceptive awareness versus accuracy (i.e., the text starting with “Somatic or interoceptive awareness centers on…” to the end of the paragraph), it should state that Chentsova-Dutton and Dzokoto (2014) were the ones who contended that awareness and accuracy are differentially driven by a distinct constellation of processes, citing evidence that awareness is driven by cultural schemas (e.g., Rimé et al., 1990) whereas accuracy is driven more by bodily cues (e.g., Jones et al., 1987); they also cite additional evidence (e.g., from Pennebaker and Hoover, 1984; Critchley et al., 2004; Fairclough and Goodwin, 2007) that the two forces do not always go hand-in-hand. It should also state that although previous studies have used performance on a heartbeat detection task (i.e., assessment of the difference between a participants' reported heartbeats and actual heartbeats) as a measure of interoceptive awareness (e.g., Pollatos et al., 2007; Herbert et al., 2012), there is now a general consensus among interception researchers that awareness and accuracy are not the same (Bornemann et al., 2015; Farb et al., 2015; Garfinkel et al., 2015), and that while awareness is about being cognitively mindful of one's bodily states, accuracy is about whether perception of one's bodily states aligns with reality; as such, people who are high on awareness are not necessarily also high on accuracy (for review, see Ceunen et al., 2013). However, variation still exists when it comes to the definition of interoceptive awareness, and what usage of that term refers to Mehling et al. (2012) and Garfinkel et al. (2015).

The original text from the same paragraph in Part 1 stated that traditional models of African medicine emphasized the holistic, harmonious integration of mind and body (Mbiti, 1970) but should state that this was a feature of traditional African religions, correct the year of the publication to 1969, and specify that according to Mbiti, traditional African religion takes a holistic approach to “occupy[ing] the whole person” (Mbiti, 1969, p. 3); as such “no line is drawn between the spiritual and physical” (Mbiti, 1969; p. 4).

In the original text, also in Part 1, that distinguished between two forms of bodily awareness (i.e., “Additionally, a further distinction can be made between proprioceptive versus interoceptive awareness…” and the following sentence), it should state that three distinctions can be made for sensory awareness more broadly (including, but not limited to, forms of bodily awareness): between: (1) exteroception or mechanoreception—i.e., of eyes, ears, skin; (2) prioprioception—i.e., of muscles, joints, and (3) interoception—i.e., of internal organs (for review, see Cacioppo et al., 2007; see also Chentsova-Dutton and Dzokoto, 2014 and Garfinkel and Critchley, 2013). Although bodily awareness more appropriately describes prioprioception and interoception (but not exteroception), it can still include mental processes that follow from perceiving and judging external features of the body (Daubenmier, 2005; Mehling et al., 2009; Daubenmier et al., 2016).

In the original text that described cultural groups who emphasized their body in language and illness (i.e., the paragraph texts that state “A growing number of recent studies has found that many cultural groups…” from Part 1 and “In the context of physical illness…” from Part 4: Interoception and Psychopathology), it should state that Chentsova-Dutton and Dzokoto (2014) observed these trends in non-European-American contexts (i.e., among the cited studies from Papua New Guinea, aboriginal Australia, West Africa, China, and Cambodia, and among African-Americans in the U.S.). It should also be noted that in addition to these cases, many cultural groups also emphasize the body in their use of language terms. To illustrate, the Ifaluk of Micronesia do not appear to have a specific word for “emotion” but rather, related words that refer more to physical, internal experiences—for example, “niferash” refers to “the most general terms used to describe internal functioning” (Lutz, 1988, p. 92). This phenomenon that has also been observed in other languages (for review, see Pavlenko, 2008). In terms of illness, numerous studies suggest that African–Americans are more likely to experience somatic symptoms in response to depression (Brown et al., 1996; Das et al., 2006). Outside the U.S., a large-scale international study across 14 countries in five continents found similar evidence for somatic symptoms as a common feature of depression (Simon et al., 1999). Similarly elevated reports of physical symptoms like pain have also been shown in physical health contexts (for example, ethnic minorities in this country report higher levels of unrelieved pain relative to European-Americans—for review, see Shavers et al., 2010).

The original text from Part 4 that stated that Cheung (1995) argued that explanations for somatization in other cultures were made *post-hoc*, but should be corrected to state that the Cheung finding and reference was put forth and cited by Ryder et al. (2008).

## Author contributions

The author confirms being the sole contributor of this work and approved it for publication.

### Conflict of interest statement

The author declares that the research was conducted in the absence of any commercial or financial relationships that could be construed as a potential conflict of interest.
